# Trends in HIV-related mortality by sex, race, age, and region in the United States, 1999–2023: A retrospective CDC WONDER database study

**DOI:** 10.1097/MD.0000000000050986

**Published:** 2026-10-16

**Authors:** Feng Jiang, Ming-Jie Jia, Ying-Lu Ma, Fei-Qing Liang, Weiwei Zeng

**Affiliations:** a Graduate School, Guangxi University of Chinese Medicine, Nanning, Guangxi, China; b Guangxi Key Laboratory of Traditional Chinese Medicine for Immune Reconstitution in HIV/AIDS, The Second Affiliated Hospital of Guangxi University of Chinese Medicine, Nanning, Guangxi, China; c The Department of Pharmacy, The Second People’s Hospital of Longgang District, Shenzhen, Guangdong, China.

**Keywords:** age-adjusted mortality rate, CDC WONDER, HIV, joinpoint regression, mortality

## Abstract

Although antiretroviral therapy has improved human immunodeficiency virus (HIV) prognosis, mortality and demographic disparities remain critical public health issues in the United States (US). This study analyzed long-term trends in HIV-related mortality from 1999 to 2023 using Centers for Disease Control and Prevention Wide-ranging Online Data for Epidemiologic Research data. This retrospective database study analyzed death certificate data for HIV-related deaths in the US from 1999 to 2023. Crude and age-adjusted mortality rates were calculated. Joinpoint regression was used to assess trends using annual percent change (APC) and average APC (AAPC), stratified by sex, race/ethnicity, age group, census region, and urbanization level. A total of 221,831 HIV-related deaths occurred from 1999 to 2023. Annual deaths declined from 14,499 in 1999 to 4719 in 2023, representing a 67.45% reduction. The age-adjusted mortality rate decreased from 8.05 to 1.92 per 100,000 population, with an AAPC of −5.73. The decline was fastest from 2007 to 2011 (APC = −10.99) but slowed from 2019 to 2023 (APC = −2.79). Mortality rates remained higher among men, non-Hispanic Black individuals, and residents of the Southern US. Although mortality declined in both metropolitan and nonmetropolitan areas, the decline was slower in nonmetropolitan areas than in metropolitan areas (AAPC = −3.92 vs −6.54). Mortality increased among older age groups, particularly those aged 75 to 84 years. HIV-related mortality in the US decreased substantially from 1999 to 2023, but the pace of decline slowed in recent years. Persistent racial, geographic, urban–rural, and age-related disparities highlight the need for targeted interventions, improved access to HIV care, and strategies addressing the growing burden among older adults.

## 1. Introduction

Human immunodeficiency virus (HIV) infection is a retroviral disease primarily targeting cluster of differentiation 4^+^ T lymphocytes, which can progress to acquired immunodeficiency syndrome (AIDS). AIDS is characterized by severe immune system dysfunction, a marked increase in the risk of opportunistic infections, and malignancies.^[1]^ Clinically, HIV infection presents a complex array of manifestations, ranging from nonspecific flu-like symptoms during the acute phase to a prolonged asymptomatic period, eventually advancing to the AIDS stage marked by a variety of opportunistic infections (such as pneumocystis pneumonia, tuberculosis) and malignancies (such as Kaposi sarcoma, lymphoma).^[2–4]^ Since the onset of the epidemic in the 1980s, HIV/AIDS has posed a major challenge to global public health. With the advent and widespread adoption of highly active antiretroviral therapy (ART), HIV infection has shifted from a fatal disease to a manageable chronic condition.^[5]^

However, as the life expectancy of individuals living with HIV has significantly increased, there has been a corresponding shift in the disease spectrum and medical needs. HIV-infected individuals are increasingly burdened by multiple chronic comorbidities, which, though common in the general population, occur at much higher rates and at earlier ages than would be expected.^[6]^ These comorbidities include cardiovascular diseases, kidney and liver disorders, osteoporosis and fractures, endocrine and metabolic abnormalities, and certain non-AIDS-related malignancies.^[7–10]^ The factors contributing to these risks are complex and intertwined, including the persistent inflammatory state directly related to chronic HIV infection, the long-term toxic effects of ART, and the widespread presence of co-infections and unhealthy lifestyles within the HIV-infected population.^[11–18]^ This shift indicates that the current assessment of the HIV disease burden must no longer focus solely on traditional opportunistic infections but must also comprehensively address the long-term health risks and challenges related to comorbidity management.

The disease burden and mortality rates associated with HIV are not uniformly distributed. Previous studies have shown substantial disparities in HIV burden and outcomes by sex, race/ethnicity, age, socioeconomic status, and geographic region.^[19]^ These disparities are closely related to differences in healthcare access, socioeconomic conditions, structural racism, stigma, and other social determinants of health.^[20]^ Despite significant progress in antiviral treatment and prevention, without targeted efforts to address these structural disparities, it will be difficult to fully eliminate HIV as a public health threat.

In this context, understanding the long-term trends in HIV-related mortality and its dynamic changes across different subpopulations is crucial for identifying disparities, evaluating the effectiveness of interventions, and optimizing resource allocation. Notably, as treatment coverage increases, the life expectancy of HIV-infected individuals has extended, and the issue of comorbidities is becoming more pronounced. According to projections based on Centers for Disease Control and Prevention (CDC) surveillance data and the North American AIDS Cohort Collaboration on Research and Design–ProjEcting Age, multimoRbidity, and poLypharmacy model, the number of individuals receiving ART in the United States (US) will increase from 670,000 in 2020 to 908,000 by 2030, with the proportion of individuals living with ≥ 2 comorbidities rising from 63 to 70%.^[21]^ This trend suggests that future prevention and treatment strategies must evolve from solely controlling the virus to comprehensively managing comorbidities and improving quality of life.

Therefore, this study used data from the CDC Wide-ranging Online Data for Epidemiologic Research (WONDER) database to comprehensively analyze age-adjusted mortality rates (AAMRs) for HIV-related deaths in the US from 1999 to 2023. This study focused on mortality differences by sex, race/ethnicity, age group, urban–rural classification, and geographic region. Joinpoint regression was used to identify key changes in mortality trends and quantify annual and average annual percent change (APC, AAPC). This national analysis may provide evidence to inform public health planning, optimize resource allocation, and guide targeted interventions for populations with persistently high HIV-related mortality.

## 2. Methods

This study utilized the CDC WONDER database to evaluate HIV mortality rates in the US from 1999 to 2023. The mortality data were derived from death certificates of US residents. This database has been widely used in numerous studies to analyze trends in mortality for specific diseases such as pneumonia and respiratory conditions.^[22,23]^ HIV-related deaths were identified using International Classification of Diseases, Tenth Revision (ICD-10) codes B20 to B24. ICD-10 codes B20 to B24 were selected as the underlying cause of death. The analysis included individuals aged 25 years and older, and age-stratified analyses were conducted for the groups 25 to 34, 35 to 44, 45 to 54, 55 to 64, 65 to 74, and 75 to 84 years. We chose to combine all HIV-related diseases for this analysis rather than analyzing them separately because these ICD-10 codes encompass 4 distinct types of HIV disease manifestations: B20 (HIV disease resulting in infectious and parasitic diseases); B21 (HIV disease resulting in malignant neoplasms); B22 (HIV disease resulting in other specified diseases); B23 (HIV disease resulting in other conditions); and B24 (unspecified HIV disease). These codes allowed us to investigate HIV-related mortality trends from 1999 to 2023. The CDC WONDER database excludes data representing fewer than 10 individuals.^[24]^ Because this study used publicly available, de-identified data, institutional review board approval and informed consent were not required.

The CDC WONDER database stratifies the data by race/ethnicity (Non-Hispanic White, Non-Hispanic Black or African American, Non-Hispanic American Indian/Alaska Native, Non-Hispanic Asian/Pacific Islander, and Hispanic/Latino),10-year age groups (25–34, 35–44, 45–54, 55–64, 65–74, and 75–84 years), urban–rural classification (using the National Center for Health Statistics urban–rural classification scheme), and census regions (Northeast, Midwest, South, and West), as defined by the US Census Bureau.^[25,26]^ For the urban–rural classification, the National Center for Health Statistics categorizes counties according to metropolitan and nonmetropolitan status.^[27]^ Metropolitan counties included large central metropolitan, large fringe metropolitan, medium metropolitan, and small metropolitan counties. Nonmetropolitan counties included micropolitan and noncore counties.^[28]^ Census regions were defined according to the US Census Bureau classification as Northeast, Midwest, South, and West.

AAMRs and crude mortality rates were calculated for each demographic group. The AAMR was standardized using the 2000 US Standard Population. We reported overall trends, as well as AAMRs for subpopulations based on sex, race/ethnicity, region, and urban–rural classification to facilitate comparisons across populations with different age structures. For age-specific analyses, crude mortality rates rather than AAMRs were reported, because age adjustment is not applicable within fixed age strata. The Joinpoint regression program (Joinpoint version 4.9.0.0, provided by the National Cancer Institute) was used to determine mortality trends from 1999 to 2023. The Monte Carlo permutation test was used to calculate the APC and AAPC, along with their 95% confidence intervals (CIs). If the slope describing the mortality change across time intervals significantly differed from zero (using a 2-tailed *t* test), the APC and AAPC were considered to indicate an increasing or decreasing trend. Statistical significance was set at *P* ≤ .05, with significance indicated by an asterisk (*).

## 3. Results

### 3.1. Overall trends

From 1999 to 2023, the total number of deaths declined from 14,499 to 4719, representing a reduction of 67.45%. The AAMR decreased from 8.05 (95% CI: 7.92–8.18) to 1.92 (95% CI: 1.86–1.98). The AAPC was −5.73 (95% CI: −6.29–−5.17), indicating a significant downward trend in mortality.

Segmented analysis revealed that from 1999 to 2003, HIV-related mortality showed a declining trend (APC = −2.63, 95% CI: −3.71–−1.53). From 2003 to 2007, the rate of decline intensified (APC = −5.81, 95% CI: −7.59–−4.00). Between 2007 and 2011, the rate of decline reached its fastest pace (APC = −10.99, 95% CI: −12.96–−8.98). From 2011 to 2019, the decline slowed (APC = −5.95, 95% CI: −6.64–−5.26). Finally, from 2019 to 2023, the APC was −2.79 (95% CI: −4.68–−0.86), with the decline further decelerating. Detailed results are presented in Table 1 and Figure 1.

**Table 1:** Trends in HIV AAMRs stratified by sex in the US, 1999–2023.

Sex	Deaths_1999	Deaths_2023	Percent change	1999 AAMR	2023 AAMR	AAPC (95% CI)
Both	14,499	4719	−67.45	8.05 (7.92–8.18)	1.92 (1.86–1.98)	−5.73 (−6.29–−5.17)*
Female	3422	1144	−66.57	3.72 (3.60–3.85)	0.93 (0.87–0.99)	−5.69 (−6.50–−4.87)*
Male	11,077	3575	−67.73	12.52 (12.28–12.75)	2.94 (2.84–3.04)	−5.73 (−6.35–−5.11)*

AAMR = age-adjusted mortality rate, AAPC = average annual percent change, CI = confidence interval, HIV = human immunodeficiency virus, US = United States.

* Indicates that the *P* value is statistically significant.

**Figure 1.**
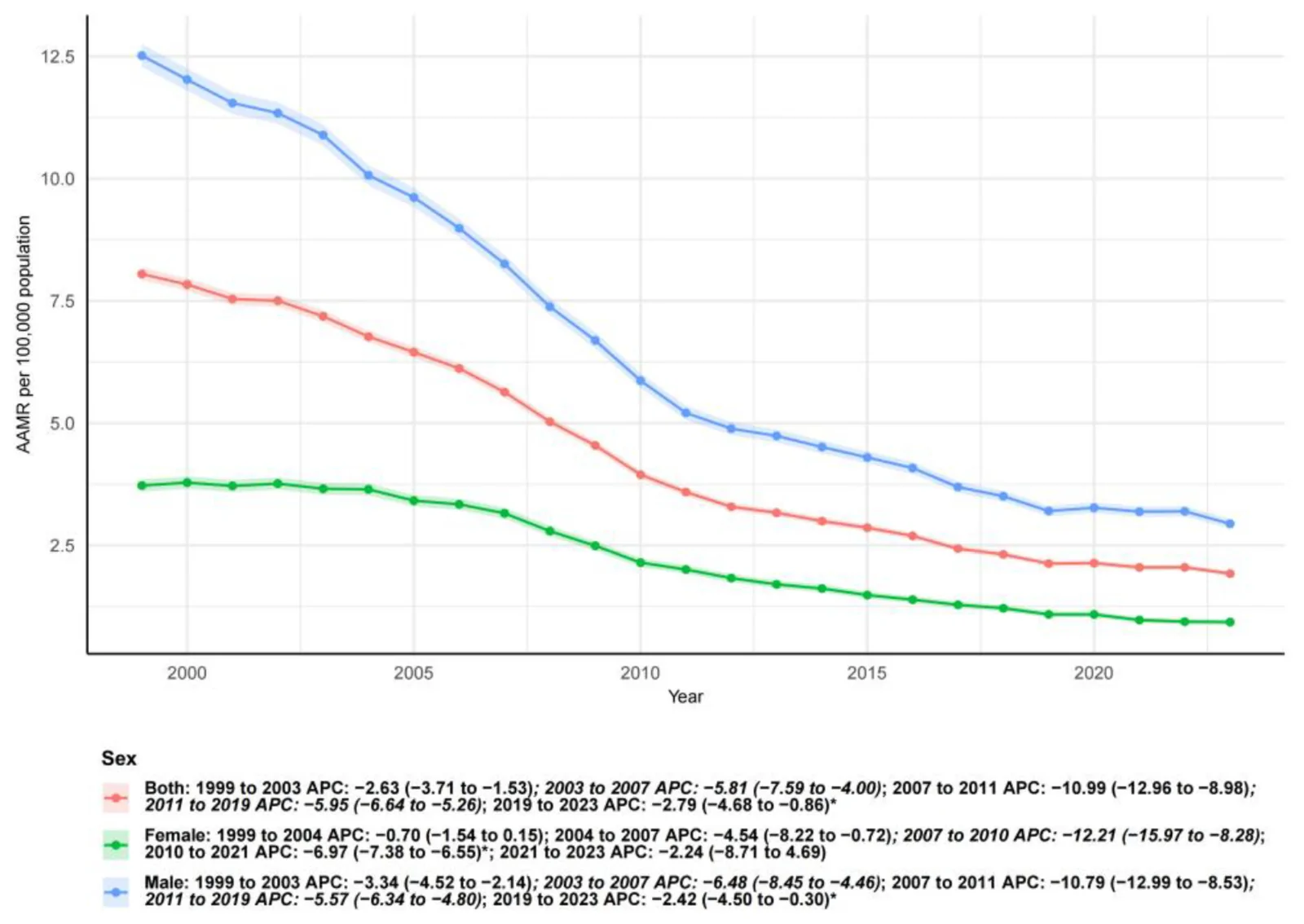
Trends in HIV AAMRs stratified by sex in the US, 1999–2023. AAMR = age-adjusted mortality rate, APC = annual percent change, HIV = human immunodeficiency virus, US = United States.

### 3.2. Demographic ddifferences

#### 3.2.1. Sex stratification

Overall, between 1999 and 2023, HIV infection in the US resulted in 164,170 male deaths (74.0%) and 57,661 female deaths (26.0%). Both male and female mortality rates showed a downward trend, with male mortality consistently higher than female mortality.

The AAMR for women decreased from 3.72 (95% CI: 3.60–3.85) in 1999 to 0.93 (95% CI: 0.87–0.99) in 2023. Segmental analysis revealed that the most significant decline in female mortality occurred between 2007 and 2010 (APC = −12.21; 95% CI: −15.97–−8.28), while from 2010 to 2021, the rate of decline slowed (APC = −6.97; 95% CI: −7.38–−6.55). From 2021 to 2023, the change was not statistically significant (APC = −2.24; 95% CI: −8.71–4.69).

For men, the AAMR decreased from 12.52 (95% CI: 12.28–12.75) in 1999 to 2.94 (95% CI: 2.84–3.04) in 2023. Segmental analysis indicated that male mortality declined most rapidly between 2007 and 2011 (APC = −10.79; 95% CI: −12.99–−8.53), followed by a slower decline from 2011 to 2019 (APC = −5.57; 95% CI: −6.34–−4.80). From 2019 to 2023, the rate of decline further decelerated (APC = −2.42; 95% CI: −4.50–−0.30). Throughout the study period, the AAPC for both sexes was nearly identical, with men at −5.73 (95% CI: −6.35–−5.11) and women at −5.69 (95% CI: −6.50–−4.87). Detailed data can be found in Table 1 and Figure 1.

#### 3.2.2. Racial stratification

Overall, the non-Hispanic Black population had the highest HIV-related mortality rate throughout the study period. HIV mortality rates showed a significant downward trend across all racial groups, with the most rapid declines observed among Hispanic and non-Hispanic Black populations between 2003 and 2013. Specific details are presented in Figure 2 and Table 2.

**Table 2:** Trends in HIV AAMRs stratified by race in the US, 1999–2023.

Race	1999 Deaths	2023 Deaths	Percent change	1999 AAMR	2023 AAMR	AAPC (95% CI)
Hispanic	1908	742	−61.11	10.59 (10.09–11.09)	1.99 (1.84–2.13)	−6.65 (−7.52–−5.77)*
NH Black	7554	2239	−70.36	36.41 (35.58–37.23)	7.64 (7.31–7.96)	−6.22 (−6.73–−5.70)*
NH White	4691	1572	−66.49	3.57 (3.47–3.68)	0.98 (0.92–1.03)	−5.21 (−6.15–−4.25)*
NH Other	142	112	−21.13	1.63 (1.36–1.90)	0.56 (0.45–0.66)	−4.64 (−5.30–−3.98)*

AAMR = age-adjusted mortality rate, AAPC = average annual percent change, CI = confidence interval, HIV = human immunodeficiency virus, NH = non-Hispanic, US = United States.

* Indicates that the *P* value is statistically significant.

**Figure 2.**
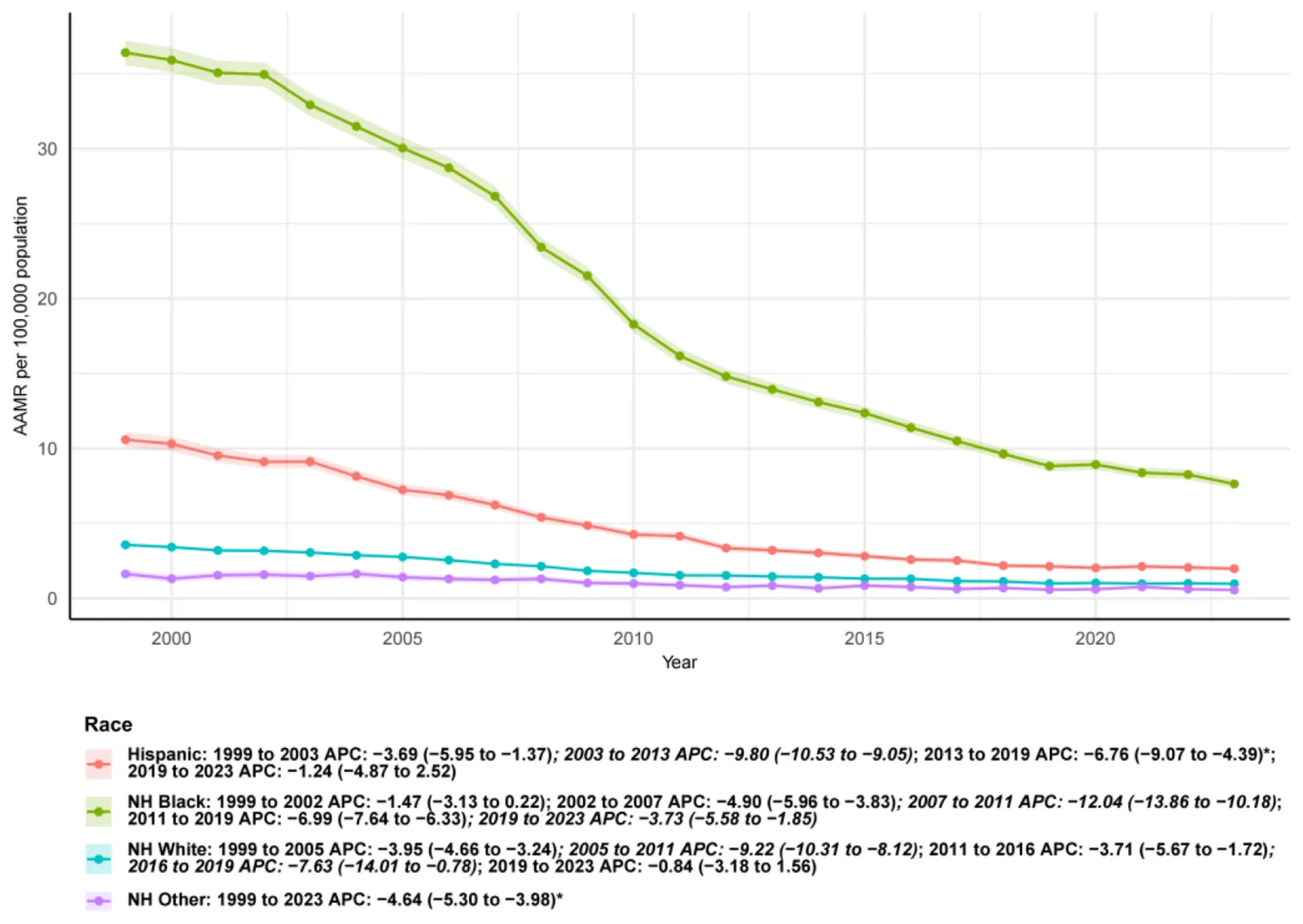
Trends in HIV AAMRs stratified by race in the US, 1999–2023. AAMR = age-adjusted mortality rate, APC = annual percent change, HIV = human immunodeficiency virus, NH = non-Hispanic, US = United States.

From 1999 to 2023, there were 30,058 HIV-related deaths among Hispanics. The AAMR for this group decreased from 10.59 (95% CI: 10.09–11.09) in 1999 to 1.99 (95% CI: 1.84–2.13) in 2023. Mortality rates showed a significant decline between 1999 and 2003 (APC = −3.69; 95% CI: −5.95–−1.37), with the steepest decline observed from 2003 to 2013 (APC = −9.80; 95% CI: −10.53–−9.05). From 2013 to 2019, the rate of decline slowed (APC = −6.76; 95% CI: −9.07–−4.39), and from 2019 to 2023, no significant change was observed (APC = −1.24; 95% CI: −4.87–2.52). Over the entire study period, the mortality rate showed a significant decline (AAPC = −6.65; 95% CI: −7.52–−5.77).

The non-Hispanic Black population experienced 118,242 HIV-related deaths. The AAMR for this group decreased from 36.41 (95% CI: 35.58–37.23) in 1999 to 7.64 (95% CI: 7.31–7.96) in 2023. Mortality rates showed no significant decline from 1999 to 2002 (APC = −1.47; 95% CI: −3.13–0.22), followed by a significant decrease between 2002 and 2007 (APC = −4.90; 95% CI: −5.96–−3.83). The rate of decline was fastest from 2007 to 2011 (APC = −12.04; 95% CI: −13.86 to −10.18), after which the decline slowed from 2011 to 2019 (APC = −6.99; 95% CI: −7.64–−6.33), and from 2019 to 2023, the rate of decline further decelerated (APC = −3.73; 95% CI: −5.58–−1.85). Overall, the mortality rate showed a significant downward trend across the study period (AAPC = −6.22; 95% CI: −6.73–−5.70).

For the non-Hispanic White population, there were 68,183 HIV-related deaths. The AAMR decreased from 3.57 (95% CI: 3.47–3.68) in 1999 to 0.98 (95% CI: 0.92–1.03) in 2023. Mortality rates significantly declined between 1999 and 2005 (APC = −3.95; 95% CI: −4.66–−3.24), accelerated from 2005 to 2011 (APC = −9.22; 95% CI: −10.31–−8.12), slowed between 2011 and 2016 (APC = −3.71; 95% CI: −5.67–−1.72), and accelerated again from 2016 to 2019 (APC = −7.63; 95% CI: −14.01–−0.78). From 2019 to 2023, no significant change was observed (APC = −0.84; 95% CI: −3.18–1.56). Over the entire period, the mortality rate showed a significant decline (AAPC = −5.21; 95% CI: −6.15–−4.25).

Among the non-Hispanic other population, there were 3152 HIV-related deaths. The AAMR decreased from 1.63 (95% CI: 1.36–1.90) in 1999 to 0.56 (95% CI: 0.45–0.66) in 2023. This group maintained a consistent downward trend throughout the study period (APC = −4.64; 95% CI: −5.30–−3.98), with an AAPC of −4.64 (95% CI: −5.30–−3.98).

#### 3.2.3. Age stratification

In the 25 to 34 age group, there were 25,333 deaths, with the crude mortality rate decreasing from 6.79 (95% CI: 6.54–7.05) in 1999 to 0.96 (95% CI: 0.87–1.05) in 2023. Mortality rates in this age group declined most rapidly between 1999 and 2011 (APC = −11.06; 95% CI: −11.47–−10.65), slowed between 2011 and 2017 (APC = −6.53; 95% CI: −9.01–−3.99), and showed no significant change from 2017 to 2023 (APC = −1.46; 95% CI: −3.68–0.82). Throughout the study period, mortality rates showed a significant downward trend (AAPC = −7.61; 95% CI: −8.39–−6.83). Detailed data are shown in Figure 3 and Table 3.

**Table 3:** Trends in HIV crude mortality rates stratified by age group in the US, 1999–2023.

Age	Deaths_1999	Deaths_2023	Percent change	Crude rate_1999	Crude rate_2023	AAPC (95% CI)
25–34 yrs	2729	436	−84.02	6.79 (6.54–7.05)	0.96 (0.87–1.05)	−7.61 (−8.39–−6.83)*
35–44 yrs	6232	710	−88.61	13.83 (13.48–14.17)	1.60 (1.48–1.72)	−8.38 (−9.28–−7.48)*
45–54 yrs	3907	901	−76.94	10.68 (10.35–11.02)	2.23 (2.08–2.37)	−6.32 (−6.61–−6.02)*
55–64 yrs	1149	1396	21.50	4.83 (4.55–5.11)	3.34 (3.16–3.51)	−1.71 (−2.04–−1.38)*
65–74 yrs	398	900	126.13	2.16 (1.95–2.37)	2.59 (2.43–2.76)	0.63 (0.35–0.90)*
75–84 yrs	70	318	354.29	0.57 (0.45–0.72)	1.73 (1.54–1.92)	4.09 (3.70–4.48)*

AAPC = average annual percent change, CI = confidence interval, HIV = human immunodeficiency virus, US = United States.

* Indicates that the *P* value is statistically significant.

**Figure 3.**
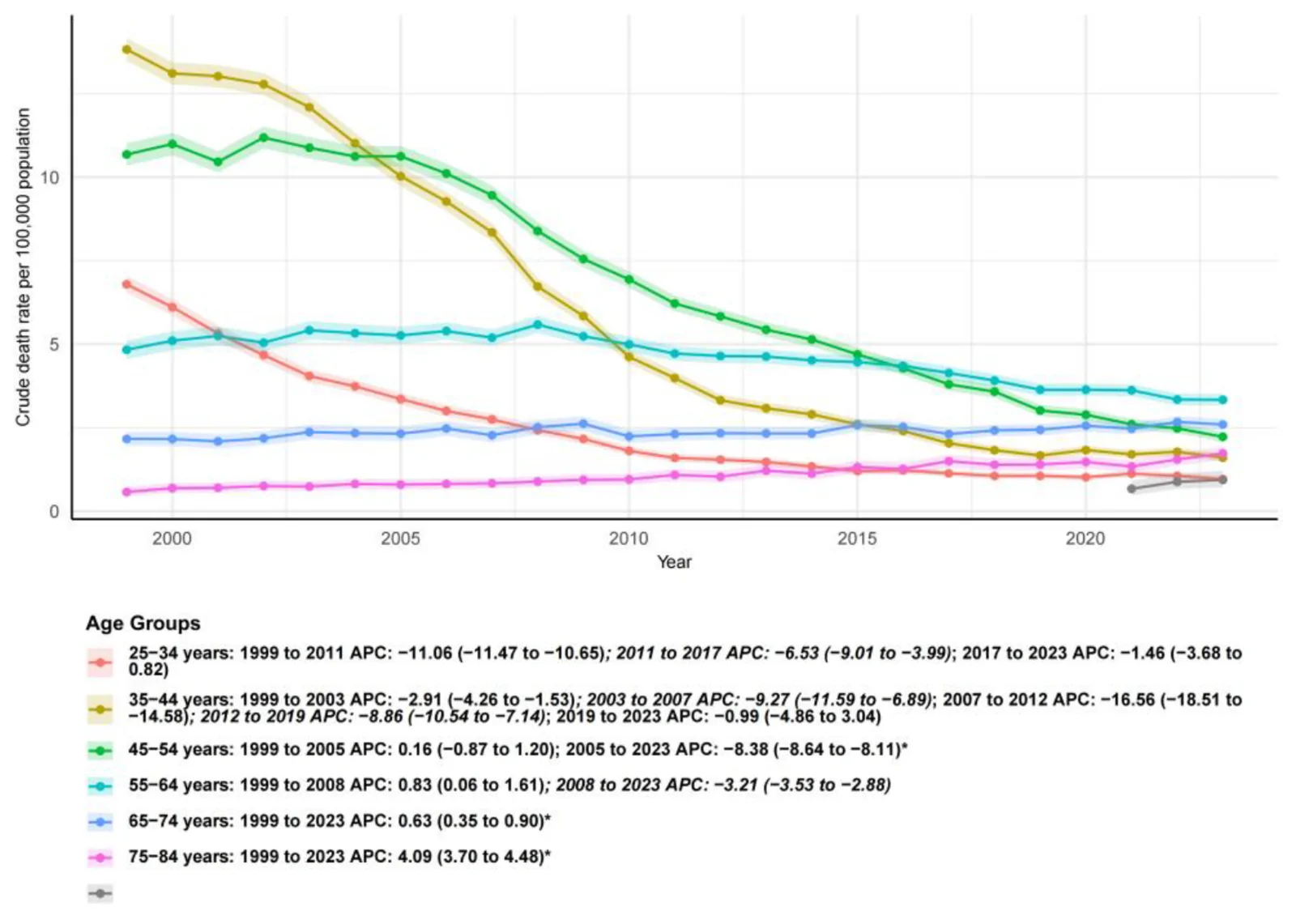
Trends in HIV crude mortality rates stratified by age group in the US, 1999–2023. APC = annual percent change, HIV = human immunodeficiency virus, US = United States.

In the 35 to 44 age group, there were 65,680 deaths, and the crude mortality rate decreased from 13.83 (95% CI: 13.48–14.17) in 1999 to 1.60 (95% CI: 1.48–1.72) in 2023. Mortality rates significantly declined between 1999 and 2003 (APC = −2.91; 95% CI: −4.26–−1.53), accelerated between 2003 and 2007 (APC = −9.27; 95% CI: −11.59–−6.89), and decreased most steeply between 2007 and 2012 (APC = −16.56; 95% CI: −18.51–−14.48). From 2012 to 2019, the rate of decline slowed (APC = −8.86; 95% CI: −10.54–−7.14), and from 2019 to 2023, no significant change was observed (APC = −0.99; 95% CI: −4.86–3.04). The overall mortality rate showed a significant downward trend throughout the study period (AAPC = −8.38; 95% CI: −9.28–−7.48).

In the 45 to 54 age group, there were 71,087 deaths, and the crude mortality rate decreased from 10.68 (95% CI: 10.35–11.02) in 1999 to 2.23 (95% CI: 2.08–2.37) in 2023. The mortality rate showed no significant change between 1999 and 2005 (APC = 0.16; 95% CI: −0.87–1.20), but significantly declined between 2005 and 2023 (APC = −8.38; 95% CI: −8.64–−8.11). Throughout the study period, the mortality rate showed a significant decline (AAPC = −6.32; 95% CI: −6.61–−6.02).

In the 55 to 64 age group, there were 40,443 deaths, and the crude mortality rate decreased from 4.83 (95% CI: 4.55–5.11) in 1999 to 3.34 (95% CI: 3.16–3.51) in 2023. Mortality rates in this group increased slightly between 1999 and 2008 (APC = 0.83; 95% CI: 0.06–1.61), followed by a significant decline from 2008 to 2023 (APC = −3.21; 95% CI: −3.53–−2.88). Throughout the study period, the mortality rate showed a significant downward trend (AAPC = −1.71; 95% CI: −2.04–−1.38).

In the 65 to 74 age group, there were 14,740 deaths, and the crude mortality rate increased from 2.16 (95% CI: 1.95–2.37) to 2.59 (95% CI: 2.43–2.76) during the study period. This group showed a significant upward trend in mortality rates throughout the study (AAPC = 0.63; 95% CI: 0.35–0.90; APC = 0.63, 95% CI: 0.35–0.90).

In the 75 to 84 age group, there were 3888 deaths, and the crude mortality rate increased from 0.57 (95% CI: 0.45–0.72) to 1.73 (95% CI: 1.54–1.92), an increase of 354.29%. This group exhibited a continuous and significant upward trend in mortality rates throughout the study period (AAPC = 4.09; 95% CI: 3.70–4.48; APC = 4.09, 95% CI: 3.70–4.48).

Overall, the trend in HIV mortality rates was highly age-dependent, with the most significant decreases observed in younger age groups (25–54 years), particularly in the 35 to 44 age group, which showed the largest reduction. In contrast, mortality rates in older age groups (65 years and above) showed an upward trend, with the rate of increase accelerating with age, especially in the 75 to 84 age group.

### 3.3. Regional differences

#### 3.3.1. Census region-based differences

Between 1999 and 2023, HIV mortality rates across different US census regions showed significant declines, but the extent and patterns of these declines varied substantially by region. The South recorded the highest number of deaths, while the Midwest had the lowest. Although the Northeast had the highest initial mortality rate, it experienced the largest decrease and the fastest annual decline rate. The Midwest, despite having lower mortality rates, exhibited stagnation in the recent decline. Both the South and West regions followed similar patterns, with initial acceleration in the decline followed by a subsequent slowdown. Detailed data are shown in Figure 4 and Table 4.

**Table 4:** Trends in HIV AAMRs stratified by census region in the US, 1999–2023.

Census Region	Deaths			AAMR		
	1999	2023	Percent change	1999	2023	AAPC (95% CI)
Northeast	4229	719	−83.00	12.05 (11.69–12.42)	1.58 (1.46–1.70)	−8.03 (−8.59–−7.46)*
Midwest	1501	551	−63.29	3.66 (3.47–3.84)	1.12 (1.02–1.22)	−4.79 (−5.76–−3.80)*
South	6549	2582	−60.57	10.24 (9.99–10.49)	2.77 (2.66–2.88)	−5.32 (−5.96–−4.66)*
West	2220	867	−60.95	5.55 (5.32–5.78)	1.51 (1.41–1.62)	−5.27 (−5.94–−4.60)*

AAMR = age-adjusted mortality rate, AAPC = average annual percent change, CI = confidence interval, HIV = human immunodeficiency virus, US = United States.

* Indicates that the *P* value is statistically significant.

**Figure 4.**
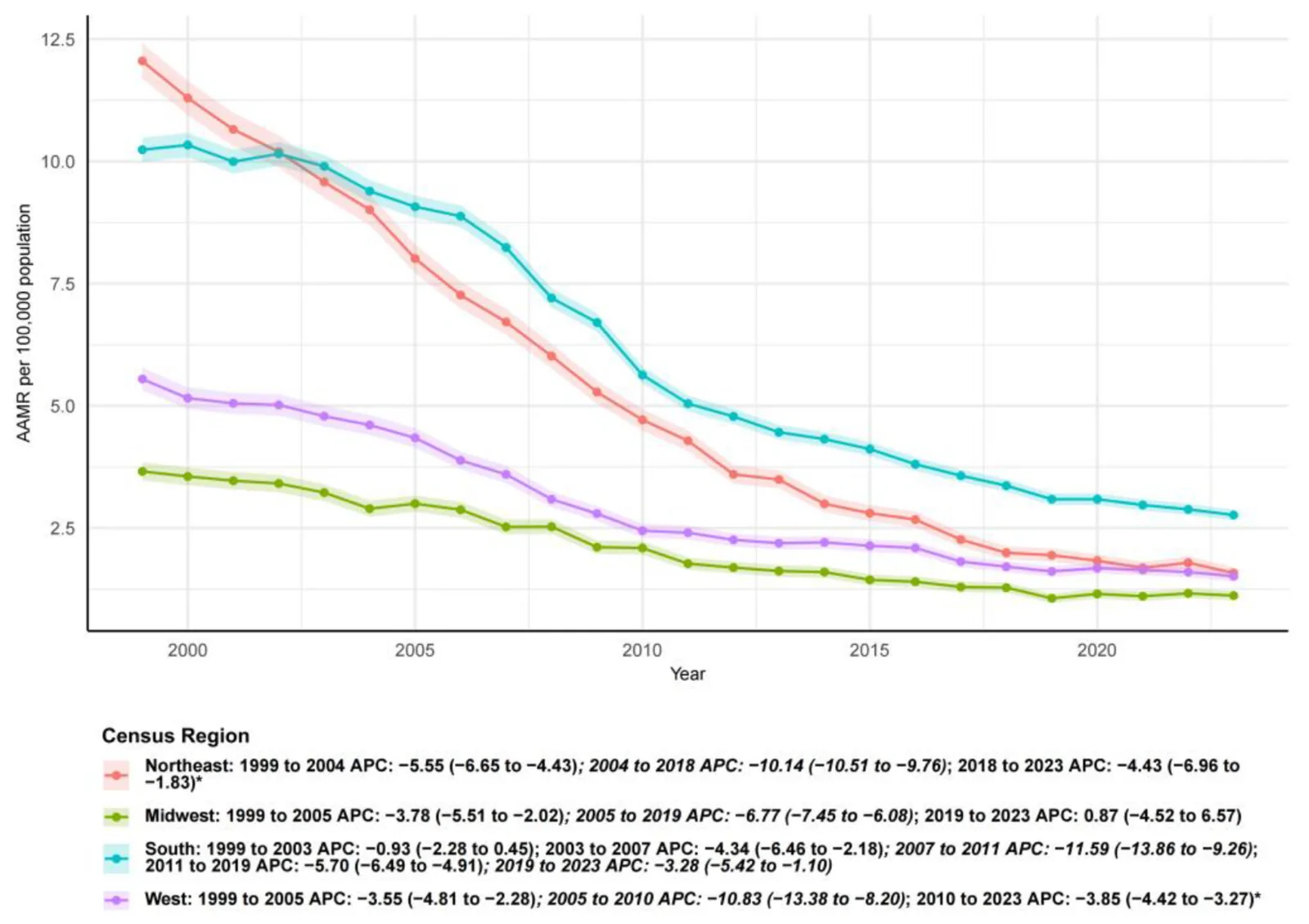
Trends in HIV AAMRs stratified by census region in the US, 1999–2023. AAMR = age-adjusted mortality rate, APC = annual percent change, HIV = human immunodeficiency virus, US = United States.

In the Northeast region, there were 50,737 deaths, with the total number of deaths decreasing from 4229 to 719, a reduction of 83%. The AAMR decreased from 12.05 (95% CI: 11.69–12.42) in 1999 to 1.58 (95% CI: 1.46–1.70) in 2023. The region saw a significant decline from 1999 to 2004 (APC = −5.55; 95% CI: −6.65–−4.43), followed by an accelerated decline between 2004 and 2018 (APC = −10.14; 95% CI: −10.51–−9.76). The decline slowed between 2018 and 2023 (APC = −4.43; 95% CI: −6.96–−1.83). Overall, the region experienced a significant decline in mortality (AAPC = −8.03; 95% CI: −8.59–−7.46).

In the Midwest region, there were 23,267 deaths, with the total number of deaths decreasing from 1501 to 551, a reduction of 63.29%. The AAMR decreased from 3.66 (95% CI: 3.47–3.84) in 1999 to 1.12 (95% CI: 1.02–1.22) in 2023. Mortality rates in this region declined significantly between 1999 and 2005 (APC = −3.78; 95% CI: −5.51–−2.02), then accelerated between 2005 and 2019 (APC = −6.77; 95% CI: −7.45–−6.08). From 2019 to 2023, no significant change was observed (APC = 0.87; 95% CI: −4.52–6.57). Throughout the study period, a significant downward trend was observed (AAPC = −4.79; 95% CI: −5.76–−3.80).

In the South region, there were 113,169 deaths, with the total number of deaths decreasing from 6549 to 2582, a reduction of 60.57%. The AAMR decreased from 10.24 (95% CI: 9.99–10.49) in 1999 to 2.77 (95% CI: 2.66–2.88) in 2023. Mortality rates in the South showed no significant change from 1999 to 2003 (APC = −0.93; 95% CI: −2.28–0.45), followed by a significant decline from 2007 to 2011 (APC = −11.59; 95% CI: −13.86–−9.26). From 2019 to 2023, the rate of decline slowed (APC = −3.28; 95% CI: −5.42–−1.10). Overall, the region exhibited a significant downward trend (AAPC = −5.32; 95% CI: −5.96–−4.66).

In the West region, there were 34,658 deaths, with the total number of deaths decreasing from 2220 to 867, a reduction of 60.95%. The AAMR decreased from 5.55 (95% CI: 5.32–5.78) in 1999 to 1.51 (95% CI: 1.41–1.62) in 2023. Mortality rates in the West declined significantly from 1999 to 2005 (APC = −3.55; 95% CI: −4.81–−2.28), followed by a rapid decline from 2005 to 2010 (APC = −10.83; 95% CI: −13.38–−8.20). From 2010 to 2023, the rate of decline slowed (APC = −3.85; 95% CI: −4.42–−3.27). Throughout the study period, the region showed a significant downward trend (AAPC = −5.27; 95% CI: −5.94–−4.60).

#### 3.3.2. State-level differences

Between 1999 and 2023, the AAMR for HIV in US states generally showed declining trends, but the magnitude of the decline varied substantially. The District of Columbia had the highest AAMR in 1999 and showed a marked decline, with its AAMR decreasing from 70.22 (95% CI: 61.64–78.80) to 10.64 (95% CI: 7.82–14.14). The fastest average annual declines were observed in New Jersey (AAPC = −8.92; 95% CI: −9.41–−8.42), New York (AAPC = −8.37; 95% CI: −8.92–−7.83), Connecticut (AAPC = −7.96; 95% CI: −9.17–−6.74), Massachusetts (AAPC = −7.75; 95% CI: −9.17–−6.32), Pennsylvania (AAPC = −7.41; 95% CI: −8.77–−6.02), Illinois (AAPC = −7.35; 95% CI: −7.90–−6.80), and the District of Columbia. Detailed results are presented in Figure 5.

**Figure 5.**
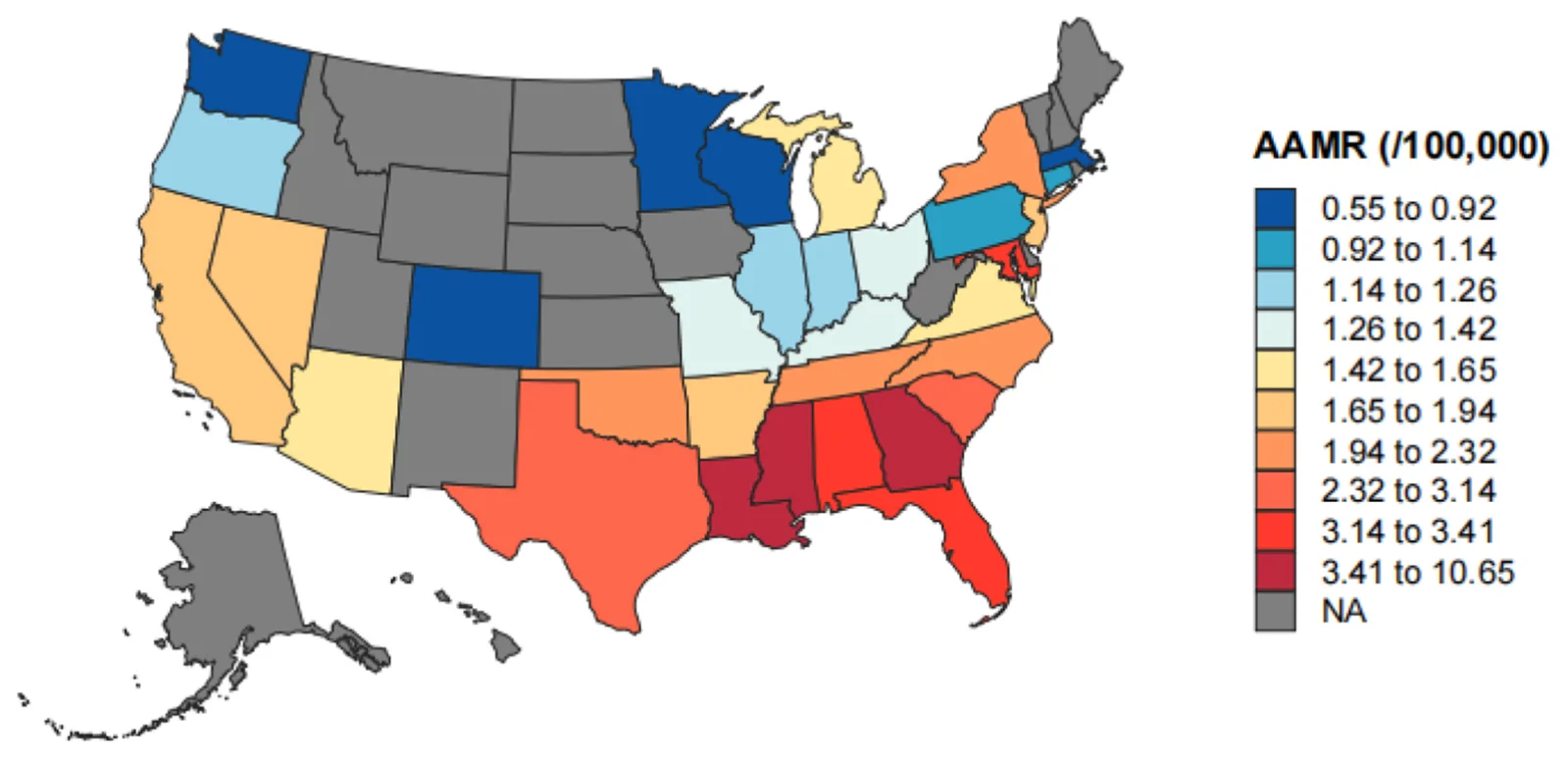
State-level AAMRs for HIV-related deaths in the US from 1999 to 2023. AAMR = age-adjusted mortality rate, HIV = human immunodeficiency virus, US = United States.

In contrast, Oregon experienced the smallest decline (AAPC = −3.68; 95% CI: −8.98–1.92), with no significant change. Oklahoma (AAPC = −2.73; 95% CI: −4.51–−0.91) and Kentucky (AAPC = −3.67; 95% CI: −4.61–−2.72) also had relatively slower declines, although statistically significant.

Most states showed declining HIV-related mortality rates during the observation period, although the magnitude and statistical significance of the decline varied by state. Among the ten states with the largest decreases, the AAPC values were statistically significant, indicating notable downward trends in these regions. Detailed data on AAMR and AAPC for each state are provided in File S1, Supplemental Digital Content 1.

#### 3.3.3. Rural vs urban differences

Overall, between 1999 and 2023, Metropolitan areas reported 191,542 HIV-related deaths, while Nonmetropolitan areas reported 15,730. Both types of regions exhibited significant declines in HIV mortality rates, but the magnitude of the decline varied noticeably. Detailed results are presented in Table 5 and Figure 6.

**Table 5:** Trends in HIV AAMRs stratified by urbanization level in the US, 1999–2023.

Urbanization	Deaths			AAMR		
	1999	2023	Percent change	1999	2023	AAPC (95% CI)
Metropolitan	13,637	4252	−68.82	8.96 (8.81–9.11)	2.25 (2.19–2.32)	−6.54 (−6.96–−6.12)*
Nonmetropolitan	862	467	−45.82	3.12 (2.91–3.33)	1.46 (1.32–1.60)	−3.92 (−4.87–−2.97)*v

The classification of urbanization levels in 2023 by AAMR uses data from 2020, and the calculation time range for AAPC is from 1999 to 2023.

AAMR = age-adjusted mortality rate, AAPC = average annual percent change, CI = confidence interval, HIV = human immunodeficiency virus, US = United States.

* Indicates that the *P* value is statistically significant.

**Figure 6.**
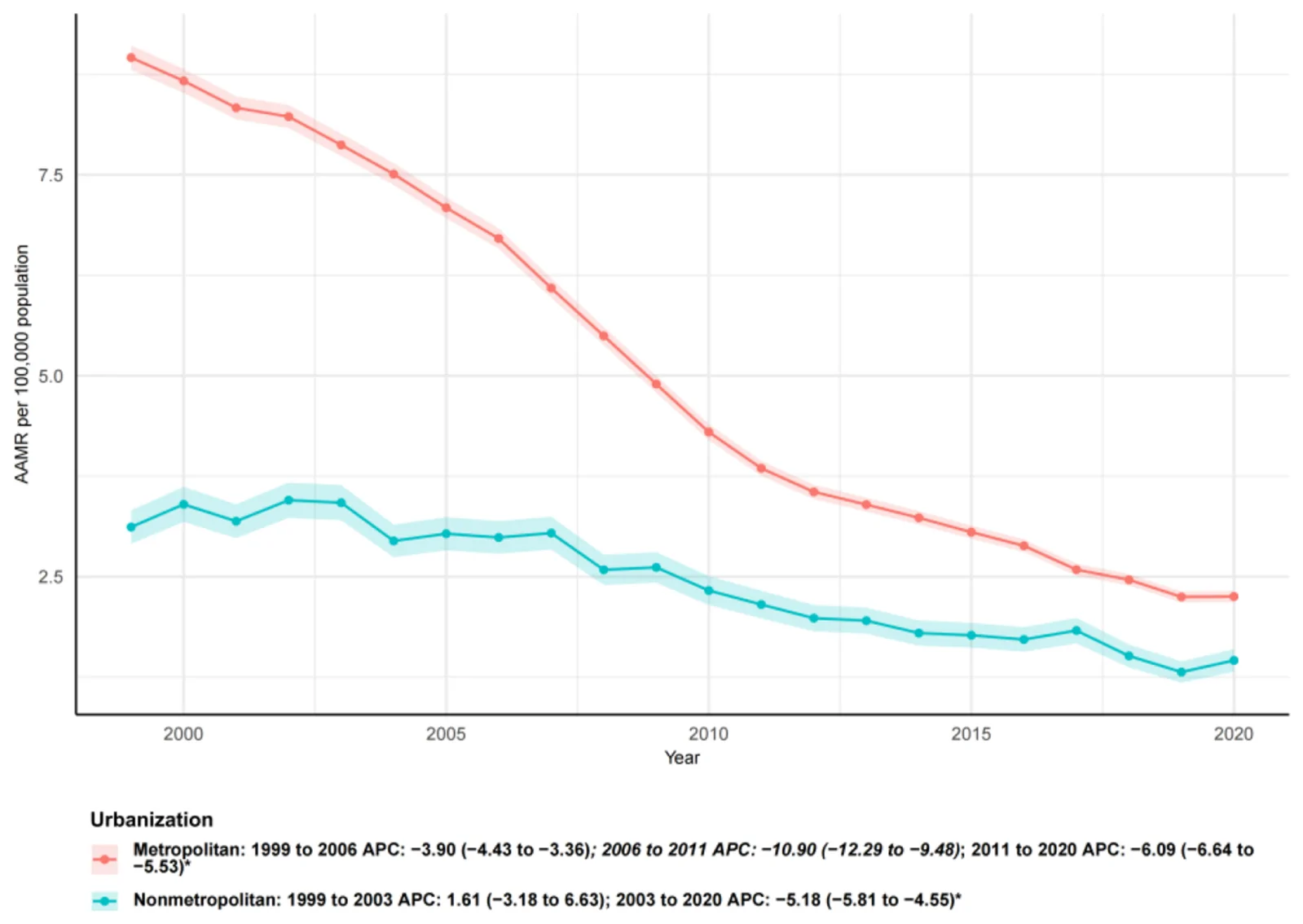
Trends in HIV AAMRs stratified by urbanization level in the US, 1999–2023. AAMR = age-adjusted mortality rate, APC = annual percent change, HIV = human immunodeficiency virus, US = United States.

In Metropolitan areas, the AAMR decreased from 8.96 (95% CI: 8.81–9.11) in 1999 to 2.25 (95% CI: 2.19–2.32) in 2023. In Nonmetropolitan areas, the AAMR decreased from 3.12 (95% CI: 2.91–3.33) to 1.46 (95% CI: 1.32–1.60). In Metropolitan areas, mortality rates significantly declined from 1999 to 2006 (APC = −3.90; 95% CI: −4.43–−3.36), with the most rapid decline observed from 2006 to 2011 (APC = −10.90; 95% CI: −12.29–−9.48). From 2011 to 2020, the decline remained steady (APC = −6.09; 95% CI: −6.64–−5.53). In Nonmetropolitan areas, the mortality rate showed no significant change from 1999 to 2003 (APC = 1.61; 95% CI: −3.18–6.63), but declined significantly from 2003 to 2020 (APC = −5.18; 95% CI: −5.81–−4.55).

Throughout the study period, both regions exhibited significant declines in mortality rates. However, the decline was more rapid in metropolitan areas (AAPC = −6.54; 95% CI: −6.96–−6.12) than in nonmetropolitan areas (AAPC = −3.92; 95% CI: −4.87–−2.97).

## 4. Discussion

This study utilized the US death surveillance database from 1999 to 2023 to comprehensively evaluate the long-term trends, demographic, and geographic distribution differences in HIV-related mortality. The results demonstrate a sharp decline in the AAMR for HIV in the US over the past 24 years. This positive trend reflects significant achievements in global and US HIV prevention and control efforts, particularly in the areas of widespread highly active ART, the promotion of pre-exposure prophylaxis (PrEP), and the strengthening of public health interventions.^[29,30]^ However, our analysis further reveals that this declining trend is not evenly distributed across all subgroups. Significant racial, age, sex, regional, and urban–rural disparities highlight deep-rooted health inequities behind the HIV disease burden.^[31]^ These disparities are not incidental and may reflect the combined effects of social determinants of health, healthcare access, policy implementation, and cultural factors.^[32]^ Therefore, this discussion interprets the observed disparities, considers their public health implications, and highlights potential directions for targeted prevention and care strategies.

This study observed an overall annual APC of −5.73% in HIV-related mortality in the US, and Joinpoint regression analysis revealed distinct phase characteristics. Between 2007 and 2011, the APC reached −10.99%, representing the steepest period of decline in mortality rates and coinciding with continued improvements in and wider adoption of ART. As treatment regimens became more effective, better tolerated, and easier to use, viral suppression rates dramatically improved, not only significantly enhancing the quality of life for those infected but also fundamentally transforming HIV from a “fatal disease to a manageable “chronic illness.”^[33–35]^ During the subsequent period from 2011 to 2019, the decline slowed but remained substantial, which may reflect expanded treatment coverage and continued strengthening of HIV prevention and care systems.

However, the marked deceleration in the decline after 2019 is concerning. This may suggest that existing prevention and treatment strategies are reaching populations for whom additional engagement and care-continuity strategies are needed.^[36,37]^ The reasons for this slowdown are likely multifaceted: first, the population of undiagnosed individuals or those who have been diagnosed but failed to link to care has become a difficult-to-reach “hard-to-engage” group; second, even within treatment, issues such as poor adherence or drug resistance may persist^[38]^; third, although the promotion of PrEP has been effective, coverage within key populations still has room for improvement.^[39]^ Moreover, the coronavirus disease 2019 (COVID-19) pandemic, which began in late 2019, may have disrupted the continuity of HIV care services, including diagnostic testing, follow-up visits, and access to medications, potentially contributing to the recent deceleration.^[40,41]^ In addition to the impact of COVID-19 on the healthcare system, the prevalence of primary drug resistance (transmitted drug resistance) could be an overlooked biomedical factor. For instance, studies in the Washington, D.C. metropolitan area found that the transmitted drug resistance rate among untreated individuals was as high as 22.5%.^[42]^ Such resistance may increase the risk of delayed viral suppression or treatment failure in some patients, although the present study could not directly evaluate individual-level resistance patterns or treatment outcomes.

The decline in mortality rates was similar between males and females, although this aggregate pattern may mask sex-specific epidemiological contexts. The persistently higher mortality among males may partly reflect the historically higher HIV burden among men who have sex with men, although sexual behavior data were not available in CDC WONDER and could not be directly evaluated in this study.^[43,44]^ The decline in male mortality may have been influenced by broader ART access and biomedical prevention strategies, including PrEP and treatment as prevention, although this study could not determine the direct contribution of specific interventions.^[45,46]^ In contrast, the decline in female mortality was largely due to the massive success of maternal HIV screening and the prevention of mother-to-child transmission programs, as well as improved healthcare accessibility for women, particularly women of color.^[47,48]^ Women from racial and ethnic minority backgrounds may continue to face structural barriers, including stigma, economic vulnerability, and limited access to prevention and care services; gender identity–specific disparities could not be assessed in this study.^[49,50]^

The racial differences observed in this study highlight substantial and persistent health inequities. The AAMR among Non-Hispanic Black individuals was more than 10 fold higher than that among Non-Hispanic White individuals in 1999 and remained nearly eightfold higher in 2023, despite a substantial decline over the study period. This disparity is unlikely to be explained by biological differences and may instead reflect the effects of structural racism and unequal access to social and healthcare resources in the US.^[51,52]^ Compared to non-Hispanic Whites, non-Hispanic Blacks are more likely to face poverty, lower educational levels, housing instability, and insufficient healthcare coverage, all of which directly limit their ability to access high-quality HIV prevention, testing, and treatment services.^[53,54]^ Minority communities often lack sufficient healthcare resources, and the historical legacy of medical abuse (e.g., the Tuskegee syphilis experiment) has led to deep mistrust of the healthcare system, becoming a significant barrier to testing and care.^[55,56]^ Higher incarceration rates may disrupt social networks, continuity of care, employment, and housing stability after release, thereby worsening access to HIV prevention and treatment services.^[57]^ Hispanic populations face similar but somewhat less severe challenges, including language barriers, immigration status issues, and limited access to healthcare. These findings suggest that biomedical interventions alone may be insufficient to eliminate racial disparities in HIV outcomes without parallel efforts to address structural and social determinants of health.^[58,59]^

An essential epidemiological shift was observed in the study: the HIV mortality burden is clearly shifting toward older populations.^[60]^ The 25 to 54 age group showed the most rapid decline in mortality, largely due to strengthened sexual health education, active promotion of testing, and early treatment interventions.^[61]^ However, mortality increased among adults aged 65 years and older, particularly those aged 75 to 84 years, representing an emerging public health concern. One possible explanation is the extended life expectancy of people living with HIV, which has led to a growing population of older adults living with HIV.^[62]^ Older HIV-infected individuals not only face the effects of HIV itself but also experience the accelerated onset of non-AIDS-defining diseases, such as cardiovascular diseases, cancer, neurocognitive disorders, and osteoporosis. Polypharmacy increases the complexity of drug interactions.^[63,64]^ Healthcare providers and older individuals themselves often lack awareness of the risks of HIV in older populations, assuming that they are sexually inactive, leading to delayed diagnoses and late-stage disease at the time of diagnosis.^[65]^ Age-related stigma and loneliness also hinder testing and discussions around sexual health.^[66]^ This shift requires the healthcare system to move from a model centered on middle-aged and young individuals to one that integrates geriatrics with HIV care, in response to the growing demand for comorbidity management and long-term care.^[67]^

Regional and state-level differences in mortality trends may provide indirect insight into the effects of differences in public health infrastructure, policy implementation, and resource allocation. The Northeast region, an early epicenter of the epidemic, experienced the fastest decline, which may be related to stronger healthcare infrastructure, public health funding, Medicaid expansion, and state-level HIV programs. These factors may have collectively promoted access to and utilization of HIV services.^[68]^ In contrast, the South recorded the highest number of deaths and had a slower decline, which may be related to differences in Medicaid expansion, poverty, rural residence, healthcare access, and HIV-related stigma.^[69]^ The stagnation in the decline observed in the Midwest (2019–2023) is particularly concerning, possibly signaling insufficient resource allocation or ineffective interventions in key populations. Rural-urban differences further exacerbated health inequities.^[70]^ Metropolitan areas showed a more rapid decline than nonmetropolitan areas, with rural areas facing multidimensional challenges: limited infectious disease specialists, pharmacies, and testing sites, requiring long travel to access services.^[71]^ Tight-knit community structures made it difficult to ensure privacy when seeking HIV-related services, and high stigma prevented many from seeking care.^[72]^ Limited broadband access hampered the use of telemedicine, which has become an important means of consultation and care, particularly during and after the COVID-19 pandemic.^[73]^ These underserved areas may benefit from innovative approaches, such as mobile testing units, expanded community health worker models, and strengthened primary care capacity for HIV prevention and treatment.^[74]^

The findings of this study have clear public health policy implications. To sustain mortality reductions and reduce disparities, several public health strategies should be considered: first, targeted interventions for underserved populations: increase outreach efforts for populations with historically high HIV burden, such as men who have sex with men, transgender individuals, sex workers, and people who inject drugs, while recognizing that these groups could not be directly evaluated in the present CDC WONDER analysis. Promote HIV self-testing and use partner services and social network strategies to identify undiagnosed individuals.^[75,76]^ Second, address structural determinants and racism: invest in underserved communities and address poverty, education, housing instability, and healthcare access.^[77,78]^ Expand Medicaid coverage in all states as the most direct way to improve healthcare access.^[79]^ Conduct anti-racism training within healthcare systems and establish culturally competent healthcare environments.^[80]^ Third, build an age-friendly HIV care system: train healthcare providers to improve their awareness of HIV risks in older adults.^[81]^ Incorporate comprehensive geriatric assessments into routine HIV care to manage comorbidities and polypharmacy.^[82]^ Launch health education campaigns targeting older populations to break down age-related stigma around sexual health.^[83]^ Fourth, bridge the regional and rural-urban gaps: increase public health funding for Southern and Midwest states. Promote integrated services in rural areas by combining HIV prevention and treatment with primary care, mental health, and substance abuse services.^[84]^ Promote the development of telemedicine to address geographic isolation issues.^[85]^ Fifth, embrace technological innovation: use big data and geographic information systems to accurately locate service gaps and high-risk areas.^[86]^ Develop digital health tools (e.g., apps) for patient education, medication reminders, and remote follow-ups.^[87,88]^

Similar to our study, Ekpendu et al analyzed trends in HIV-related mortality in the US from 1999 to 2023 using the same CDC WONDER database, ICD-10 codes (B20–B24), and Joinpoint regression.^[89]^ The core conclusions of the 2 studies are consistent: HIV-related mortality declined substantially overall but remained disproportionately high among males, Non-Hispanic Black individuals, and residents of the South, with the mortality burden shifting toward older populations, findings that further support the credibility of our results. Several differences are noteworthy. First, Ekpendu et al reported a higher cumulative number of deaths (271,932) and a smaller overall AAPC (−4.34%) than the present study (221,831; −5.73%), likely because we restricted our analysis to individuals aged ≥ 25 years. Second, whereas Ekpendu et al noted higher absolute mortality rates in large metropolitan areas, our study found that the decline in mortality was markedly slower in nonmetropolitan areas than in metropolitan areas (AAPC −3.92 vs −6.54); these findings reflect different dimensions (the former the absolute burden attributable to case concentration, and the latter the unequal pace of improvement over time) and are therefore not contradictory. Third, our study identified a sustained upward trend in mortality among adults aged 75 to 84 years, complementing the emphasis of Ekpendu et al on the persistently high burden among those aged 45 to 54 years. In addition, Ekpendu et al incorporated the county-level Social Vulnerability Index and demonstrated its independent association with HIV mortality, whereas the incremental contributions of the present study include state-level Joinpoint analyses, more granular urban–rural comparisons, and precise characterization of key inflection points. Overall, the 2 studies are complementary and jointly underscore the central challenge that gains in HIV prevention and treatment remain unevenly distributed across population subgroups.

This study also has several limitations. First, death certificate data may contain coding errors or misclassifications. Second, we were unable to obtain individual-level data on behaviors, socioeconomic status, or treatment adherence, limiting our ability to conduct deeper mechanistic analyses of observed disparities. Third, the Joinpoint model primarily reflects statistical trend changes, and the interpretation of its breakpoints requires consideration of historical events. Finally, the data for 2023 may still be affected by the ongoing impact of the COVID-19 pandemic, with its long-term effects requiring further monitoring.

## 5. Conclusion

This study demonstrates that HIV-related mortality in the US declined substantially from 1999 to 2023. However, the pace of decline slowed after 2011 and further decelerated from 2019 to 2023. These recent changes coincided with the COVID-19 pandemic, which may have disrupted HIV testing, prevention, follow-up, and treatment services. Substantial disparities persisted across population groups. Mortality rates remained disproportionately high among men, non-Hispanic Black individuals, and residents of the South, while the decline was slower in nonmetropolitan than metropolitan areas. Of particular concern, mortality increased among adults aged 65 years and older, especially those aged 75 to 84 years. These findings suggest that although major progress has been achieved in HIV prevention and treatment, future public health strategies should focus on sustaining mortality reductions, improving access to HIV care, addressing racial and geographic disparities, and adapting HIV services to the growing needs of older adults living with HIV.

## Acknowledgments

We are grateful to Professor Weiwei Zeng for the financial support.

## Author contributions

**Conceptualization:** Ming-Jie Jia, Ying-Lu Ma, Weiwei Zeng.

**Data curation:** Ming-Jie Jia, Ying-Lu Ma.

**Funding acquisition:** Weiwei Zeng.

**Methodology:** Weiwei Zeng.

**Resources:** Fei-Qing Liang.

**Software:** Fei-Qing Liang.

**Supervision:** Fei-Qing Liang, Weiwei Zeng.

**Validation:** Fei-Qing Liang.

**Visualization:** Fei-Qing Liang.

**Writing – original draft:** Feng Jiang.

**Writing – review & editing:** Feng Jiang, Fei-Qing Liang, Weiwei Zeng.


