# Supplementary material for: Trends in HIV-related mortality by sex, race, age, and region in the United States, 1999–2023: A retrospective CDC WONDER database study
Source: Medicine (Baltimore). 2026 Oct 16;105(42):e50986. doi: 10.1097/MD.0000000000050986 (PMC13680913; doi:10.1097/MD.0000000000050986)
Supplement: Supplementary file 1 [file medi-105-e50986-s001.docx]

| **Measure** | **AAMR_1999** | **AAMR_2023** | **AAPC (95% CI)** |
| --- | --- | --- | --- |
| Alabama | 6.06 (5.15 to 6.97) | 3.14 (2.55 to 3.74) | -3.85 (-5.74 to -1.93)* |
| Arizona | 4.84 (4.06 to 5.62) | 1.47 (1.15 to 1.86) | -4.53 (-7.04 to -1.95)* |
| Arkansas | 4.00 (3.09 to 5.09) | 1.65 (1.14 to 2.32) | -3.46 (-5.41 to -1.47)* |
| California | 7.36 (7.00 to 7.73) | 1.94 (1.77 to 2.10) | -5.43 (-6.28 to -4.58)* |
| Colorado | 3.57 (2.88 to 4.26) | 0.85 (0.59 to 1.19) | -5.93 (-6.59 to -5.26)* |
| Connecticut | 8.82 (7.60 to 10.04) | 1.10 (0.76 to 1.54) | -7.96 (-9.17 to -6.74)* |
| District of Columbia | 70.22 (61.64 to 78.80) | 10.64 (7.82 to 14.14) | -7.32 (-8.89 to -5.73)* |
| Florida | 16.11 (15.32 to 16.90) | 3.17 (2.89 to 3.44) | -6.54 (-7.35 to -5.71)* |
| Georgia | 14.39 (13.36 to 15.41) | 3.80 (3.35 to 4.24) | -5.44 (-6.36 to -4.52)* |
| Illinois | 6.83 (6.26 to 7.41) | 1.24 (1.00 to 1.48) | -7.35 (-7.90 to -6.80)* |
| Indiana | 2.84 (2.31 to 3.37) | 1.20 (0.90 to 1.57) | -3.74 (-4.48 to -3.00)* |
| Kentucky | 2.88 (2.27 to 3.61) | 1.36 (0.98 to 1.83) | -3.67 (-4.61 to -2.72)* |
| Louisiana | 13.02 (11.68 to 14.35) | 3.54 (2.86 to 4.22) | -5.69 (-6.54 to -4.83)* |
| Maryland | 16.32 (15.00 to 17.65) | 3.21 (2.69 to 3.73) | -6.40 (-7.45 to -5.35)* |
| Massachusetts | 5.88 (5.15 to 6.62) | 0.92 (0.68 to 1.21) | -7.75 (-9.17 to -6.32)* |
| Michigan | 3.64 (3.18 to 4.11) | 1.47 (1.17 to 1.77) | -4.11 (-5.72 to -2.47)* |
| Minnesota | 2.27 (1.78 to 2.86) | 0.76 (0.52 to 1.08) | -5.14 (-6.05 to -4.22)* |
| Mississippi | 8.72 (7.33 to 10.11) | 3.41 (2.63 to 4.36) | -3.42 (-5.09 to -1.73)* |
| Missouri | 4.16 (3.49 to 4.84) | 1.29 (0.97 to 1.68) | -5.72 (-6.41 to -5.02)* |
| Nevada | 7.97 (6.42 to 9.52) | 1.88 (1.35 to 2.54) | -4.64 (-5.24 to -4.04)* |
| New Jersey | 15.96 (14.91 to 17.00) | 1.89 (1.56 to 2.23) | -8.92 (-9.41 to -8.42)* |
| New York | 18.97 (18.20 to 19.74) | 2.32 (2.07 to 2.57) | -8.37 (-8.92 to -7.83)* |
| North Carolina | 8.61 (7.81 to 9.41) | 2.25 (1.92 to 2.58) | -5.66 (-6.87 to -4.43)* |
| Ohio | 3.33 (2.91 to 3.75) | 1.34 (1.09 to 1.59) | -4.51 (-5.17 to -3.85)* |
| Oklahoma | 4.71 (3.79 to 5.63) | 2.27 (1.72 to 2.94) | -2.73 (-4.51 to -0.91)* |
| Oregon | 3.36 (2.64 to 4.21) | 1.26 (0.90 to 1.71) | -3.68 (-8.98 to 1.92) |
| Pennsylvania | 6.16 (5.61 to 6.71) | 1.14 (0.93 to 1.35) | -7.41 (-8.77 to -6.02)* |
| South Carolina | 11.77 (10.45 to 13.10) | 2.40 (1.92 to 2.95) | -5.83 (-8.21 to -3.38)* |
| Tennessee | 6.92 (6.07 to 7.77) | 2.18 (1.75 to 2.61) | -5.04 (-6.43 to -3.62)* |
| Texas | 7.95 (7.47 to 8.44) | 2.75 (2.52 to 2.98) | -4.33 (-5.47 to -3.17)* |
| Virginia | 5.57 (4.89 to 6.24) | 1.42 (1.14 to 1.76) | -5.30 (-6.86 to -3.71)* |
| Washington | 2.79 (2.27 to 3.32) | 0.92 (0.69 to 1.19) | -4.48 (-6.60 to -2.31)* |
| Wisconsin | 1.82 (1.40 to 2.33) | 0.56 (0.34 to 0.85) | -5.67 (-6.50 to -4.83)* |
